# Urinary tract infection and indwelling urinary catheters: prospective study in gynecological surgery with antibiotic prophylaxis: Infecção do trato urinário e cateter vesical de demora: um estudo prospectivo em cirurgia ginecológica com antibioticoprofilaxia

**DOI:** 10.1590/1516-3180.2014.9071412

**Published:** 2015-10-09

**Authors:** José Carlos Carraro-Eduardo, Daniela da Silva Alves, Ingrid Ellis Hinden, Ivan Penaloza Toledano, Sarah Gomes Freitas, Pedro Juan José Mondino, José Rodrigo de Moraes, Carlos Augusto Faria

**Affiliations:** I MD, MSc, PhD. Associate Professor, Department of Internal Medicine, School of Medicine, Universidade Federal Fluminense (UFF), Niterói, Rio de Janeiro, Brazil.; II Undergraduate Medical Student, Department of Internal Medicine, School of Medicine, Universidade Federal Fluminense (UFF), Niterói, Rio de Janeiro, Brazil.; III MD, MSc. Adjunct Professor, Department of Pathology, School of Medicine, Universidade Federal Fluminense (UFF), Niterói, Rio de Janeiro, Brazil.; IV MSc, PhD. Adjunct Professor, Statistics Department, Institute of Mathematics and Statistics, Universidade Federal Fluminense (UFF), Niterói, Rio de Janeiro, Brazil.; V MD. Adjunct Professor, Mother-Child Department, School of Medicine, Universidade Federal Fluminense (UFF), Niterói, Rio de Janeiro, Brazil.

**Keywords:** Urinary tract infections, Bacteriuria, Urinary catheterization, Antibiotic prophylaxis, Gynecologic surgical procedures

## Abstract

**CONTEXT AND OBJECTIVES:**

Urinary tract infections are the most common cause of hospital-acquired infections, and the use of indwelling urinary catheters is a predisposing factor for their development. The aims of this study were to estimate the frequency of pre and postoperative bacteriuria, identify the microorganisms involved, count the colony-forming units, determine the antibiotic sensitivity profile and compare the results from pre and postoperative urinalyses among women undergoing gynecological surgery with implantation of a urinary catheter.

**DESIGN AND SETTING:**

Non-controlled prospective observational single-cohort epidemiological study carried out at a university hospital.

**METHODS:**

Urine samples were collected before and 24 hours after catheterization for urinalysis, culturing and antibiotic sensitivity testing. Pre and postoperative urinalyses were compared using Wilcoxon and McNemar non-parametric tests.

**RESULTS:**

Fifty-one women participated in the study. *Escherichia coli* grew in six preoperative samples (11.8%) and *Klebsiella pneumoniae* in one (1.9%), but bacterial growth did not occur in any postoperative sample. Urinalysis showed lower number of pus cells in the postoperative urine samples (P < 0.05). There were no differences in red blood cell counts or in the nitrite and leukocyte esterase tests, between the samples.

**CONCLUSION:**

Bacteriuria was found in 13.7% of the preoperative samples. Gram-negative bacteria sensitive to most antibiotics were identified. In the postoperative samples, no bacterial growth was observed. Urinalysis only showed significant reduction of leukocyturia in the postoperative period.

## RESUMO

**CONTEXTO E OBJETIVOS:**

As infecções urinárias são a causa mais comum de infecções hospitalares, e o uso de cateteres de demora é fator predisponente para o seu desenvolvimento. Os objetivos deste estudo foram estimar a frequência de bacteriúria pré e pós-operatória, identificar os germes encontrados, a contagem de unidades formadoras de colônias e o perfil de sensibilidade aos antibióticos, além de comparar os resultados dos exames de urina pré- e pós-operatórios em mulheres submetidas a cirurgias ginecológicas com cateterismo vesical.

**TIPO DE ESTUDO E LOCAL:**

Estudo epidemiológico, observacional, de coorte única, prospectivo, não controlado, realizado em hospital universitário.

**MÉTODOS:**

Amostras de urina foram colhidas antes da cateterização e após 24 horas para urinálise, cultura e antibiograma. Os resultados da urinálise no pré- e pós-operatório foram comparados utilizando-se os testes não paramétricos de Wilcoxon e McNemar.

**RESULTADOS:**

Participaram do estudo 51 mulheres. Houve crescimento de *Escherichia coli* em seis amostras pré-operatórias (11,8%) e de *Klebsiella pneumoniae* em uma (1,9%), mas não houve crescimento bacteriano em nenhuma amostra pós-operatória. A urinálise mostrou menor quantidade de piócitos na amostra de urina pós-cirúrgica (P < 0,05). Não houve diferença quanto ao número de hemácias e às reações para nitrito e leucocitesterase, entre as amostras.

**CONCLUSÃO:**

Houve bacteriúria em 13,7% das amostras pré-operatórias, sendo identificadas bactérias Gram-negativas sensíveis à maioria dos antibióticos. Não foi observado crescimento bacteriano nas amostras pós-operatórias. A urinálise mostrou somente redução significativa da leucocitúria no pós-operatório.

## INTRODUCTION

The use of indwelling urinary catheters is the single most significant predisposing factor for the development of urinary tract infections (UTIs) in hospitalized patients, and the duration of catheterization is the most significant risk factor for this.^1^ ^3 ^UTIs occur more frequently in the postoperative period when urinary catheters are used and lead to greater morbidity and mortality, as well as increased hospitalization time and costs.^4^ ^6^

Intraoperative, pre-incision antibiotic prophylaxis is highly effective in preventing surgical site infection.^7^ In addition, some studies have suggested that antibiotic prophylaxis may also reduce the risk of catheter-related UTIs, although it is not recommended for this purpose.^8^ ^9^

Most studies on catheter-related UTIs define bacteriuria as the primary outcome; in fact, this term is often used as a synonym for UTI. However, the difference between the two is clinically relevant, since catheter-related asymptomatic bacteriuria is seldom associated with adverse outcomes and generally does not require treatment.^10^

Asymptomatic bacteriuria is defined as the presence of more than 10^5^ colony-forming units/ml (CFU/ml) of uropathogenic bacteria in a urine culture.^11^ However, in patients with urinary catheters, it is defined as the presence of more than 10^2^ CFU/ml of any one microorganism.^12^

Women who have undergone genital interventions generally receive urinary catheters for at least 24 hours. The possible correlation between preoperative asymptomatic bacteriuria and the occurrence of a UTI in such cases has not been well established, nor have the effects of intraoperative antibiotic prophylaxis on preventing UTIs. 

## OBJECTIVE

The aims of the present study, conducted with a single cohort of women who underwent elective gynecological surgery and short-term urinary catheterization, were first, to estimate the frequency of pre and postoperative bacteriuria, with identification of the microorganisms involved, counting of CFUs and determination of the antibiotic sensitivity profile, and second, to compare the results from pre and postoperative urinalyses.

## METHODS

This study was approved by the Research Ethics Committee of Fluminense Federal University. 

A prospective, observational, epidemiological, single-cohort, non-controlled study was conducted on a convenience sample from April 2010 to September 2011. Women admitted to the hospital for elective gynecological surgery including bladder catheterization were invited to participate in the study, and those who agreed signed an informed consent form. There was no sample calculation.

Participants with a diagnosis of a UTI, those using antibiotics with potential effectiveness against UTIs within 24 hours before urinary catheterization and patients younger than 18 years old were excluded from the study. 

Two urine samples were collected from each participant. The first sample was collected at the hospital ward during spontaneous urination. After transfer to the operating room, a Foley catheter was inserted following the standard technique. All participants were given pre-incision antibiotic prophylaxis with cefazolin, clindamycin or ciprofloxacin at a dose of two grams, intravenously. No participant was given further doses of antibiotics. 

The postoperative urine sample was collected directly from the drain at the bottom of the collecting bag, 24 hours after catheter placement. Any bacterial count higher than 1,000 CFUs was taken into consideration. 

The following variables were considered for analysis: age, urinalysis results (pH, specific gravity, nitrite, leukocyte esterase, hematuria and leukocyturia), urine culture results, surgical indication leading to catheterization, presence of UTI symptoms and type of antibiotic prophylaxis at surgery. 

Comparison of the pre and postoperative urinalyses of participants who exhibited bacteriuria in their preoperative sample was done using McNemar's test and the Wilcoxon signed-rank test. The significance level was set at 5% in all tests, which were performed using the Statistical Package for the Social Sciences software for Windows, version 17.0 (SPSS, Chicago, IL, USA; Microsoft, Redmond, WA, USA).

## RESULTS

A total of 61 women met the inclusion criteria, but three declined to participate, and seven were excluded because the second urine sample could not be collected. 

The participants' ages ranged from 18 to 77 years, with a mean of 48.23 ± 11.19 years of age. No participant presented symptoms of UTIs.

The antibiotics used were cefazolin, in 49 patients, and ciprofloxacin and clindamycin in one patient each. Most patients underwent abdominal surgery (47 cases), but four of them underwent vaginal surgery. The frequency of bacteriuria was 13.7% in the preoperative samples (7 cases). *Klebsiella pneumoniae* was found in 1 case and *Escherichia coli* in 6, and the CFU counts ranged from 2,000 to 100,000. The antibiotic sensitivity test revealed that all of the bacteria isolated were sensitive to the antibiotic administered for prophylaxis. Bacterial growth did not occur in any of the urine samples collected after surgery.

The specific gravities of all pre and postoperative urine samples ranged from 1,010 to 1,030, and the pH values ranged from 5 to 7.5. 

Hematuria was detected in 8 preoperative and 14 postoperative samples. Leukocyturia was detected in 8 preoperative samples, 3 of which exhibited bacterial growth, and in 9 samples collected 24 hours after catheter placement. 

The comparison of the results from the pre and postoperative urinalyses of the participants who exhibited bacteriuria in the first sample is described in Table 1. 

## DISCUSSION

In this study, the findings from urinalysis were similar to those in other reports on UTIs in the literature.^13^ ^14 ^Hematuria was detected in the preoperative samples of eight participants, and only one urine culture tested positive for bacteriuria. Since these samples were collected during spontaneous urination, the probable causes of hematuria were the clinical conditions that led to the indications of surgery, such as bleeding uterine leiomyomatosis. Hematuria was detected in the postoperative samples from 14 participants, possibly due to traumatic injury during catheter insertion. 

Our findings for nitrite did not differ from reports in the literature and exhibited low sensitivity and high specificity.^13^ It is worth noting that enterobacteria were isolated from all of the positive culture samples, since these bacteria are characteristically able to reduce nitrate to nitrite. The leukocyte esterase test was positive in 5% of the samples, although bacterial growth was detected in cultures from only 20% of them, thus indicating low sensitivity and specificity. 

There are divergent opinions regarding treatment of asymptomatic bacteriuria in patients with a urethral indwelling catheter, and antibiotic prophylaxis is not indicated for patients with long-term catheterization due to the risk of inducing microbial resistance.^12^ In addition, there is limited evidence indicating that antibiotic prophylaxis reduces the rate of bacteriuria in surgery patients who undergo postoperative urethral catheterization for at least 24 hours.^15^

**Table 1:** Pre and postoperative urinalyses of participants who exhibited bacteriuria in their preoperative samples

Bacteria isolated	Nitrite*		Leukocyte esterase^†^		Red blood cells^‡^		Pus cells^§^	
	Before	After	Before	After	Before	After	Before	After
E. coli	-	-	+	-	41	2	21	3
E. coli	-	-	-	-	1	0	5	2
E. coli	-	-	+	-	2	31	13	6
E. coli	-	-	-	-	1	3	1	1
E. coli	+	-	-	-	1	41	13	11
E. coli	-	-	-	-	0	35	2	3
Klebsiella	+	-	-	-	6	1	2	1

Before = preoperative urine sample; After = postoperative urine sample. *McNemar's test: P = 0.500; ^†^McNemar's test: P = 0.500; ^‡^Wilcoxon signed-rank test (one-tailed): P = 0.289; ^§^Wilcoxon signed-rank test (onetailed): P = 0.047.

In this study, in which the criterion used was stricter, it was found that preoperative urine cultures tested positive for bacteria sensitive to the antibiotic administered for prophylaxis in seven cases, and no bacterial growth occurred in the postoperative samples. Similarly, another study found that none of the urine samples from patients who underwent bladder catheterization during elective surgical procedures, 94% of whom were given antibiotic prophylaxis to prevent surgical wound infection, tested positive. However, the criterion used to establish the presence of a UTI was ≥ 100,000 CFU/ml of urine.^16^ Another study conducted in women who underwent gynecological surgery found that 23.6% of the urine samples collected up to 24 hours after catheter removal tested positive on culturing, with 2.4% of these patients requiring antibiotic treatment.^17^

Since the benefits of antibiotic prophylaxis to prevent surgical site infection are undeniable, it is not possible to include a control group in studies in which prophylaxis is not provided. This, together with the small sample size, is the main limitation of our study. However, the finding that none of the women with asymptomatic bacteriuria before surgery developed clinical or laboratory signs of a UTI despite urinary catheterization is important, particularly because the criterion selected for diagnosis was more sensitive (> 100 CFU/ml). Although the procedures for asepsis and the use of closed sterile collecting bags certainly contributed towards preventing the occurrence of bacterial growth in the samples collected after surgery, and although the natural history of bacteriuria in such cases is unknown, this finding might be also attributable to the antibiotic prophylaxis.

## CONCLUSIONS

The frequency of preoperative bacteriuria in this group of women undergoing gynecological surgery was 13.7%. Gram-negative bacteria sensitive to most antibiotics were identified, and the CFU counts ranged from 2,000 to 100,000. Bacterial growth did not occur in any postoperative sample. Urinalysis showed significant reduction of leukocyturia in the postoperative period, but no difference was found in the red blood cells count or in the nitrite and leukocyte esterase tests. 
